# A multi‐omics perspective of CAR T cell therapy

**DOI:** 10.1002/ctm2.1274

**Published:** 2023-05-25

**Authors:** Jingwen Yang, Yamei Chen, Leng Han

**Affiliations:** ^1^ Center for Epigenetics and Disease Prevention, Institute of Biosciences and Technology Texas A&M University Houston Texas USA; ^2^ Department of Translational Medical Sciences, College of Medicine Texas A&M University Houston Texas USA

**Keywords:** CART, Omics

## Abstract

As omics technologies, including genomics, epigenomics, transcriptomics, T cell receptor‐repertorie profiling, proteomics, metabolomics and microbiomics, have provided valuable insights into CAR T cell therapy, in our recent review, we discuss these multidimensional profiling technologies in CAR T cell research, and their potential to identify tumor‐specific antigens and molecular characteristics associated with anti‐tumour effects and toxicities.

1

Chimeric antigen receptor (CAR) T cell therapy is a novel immunotherapy that genetically modifies T cells to recognize and kill tumour cells expressing specific antigens.^1^ While CAR T cell therapy has emerged as a promising treatment option for blood malignancies, it is still challenging to optimize CAR target design, enhance CAR T cell efficacy and persistence, as well as reduce toxicity. Previous excellent reviews have summarized the progress in CAR T cell therapy, including discoveries from clinical trials, molecular mechanisms of CAR T cells, as well as considerations for patient selection for CAR T cell therapy.^2^, ^3^, ^4^ As data derived from omics technologies can provide valuable insights into different aspects of CAR T cell therapy, multidimensional omics analyses, including genomics, epigenomics, transcriptomics, T cell receptor‐repertoire profiling, proteomics, metabolomics and microbiomics, have been extensively applied in CAR T cell therapy research. In our recent published review,^5^ we provide a comprehensive overview of the multidimensional profiling technologies used in current CAR T cell research (Figure 1). Additionally, it also explores the latest research advancements and challenges associated with utilizing multidimensional omics data to identify tumour‐specific antigens and molecular characteristics that are linked to the anti‐tumour effects and toxicities of CAR T cell therapy.

**FIGURE 1:**
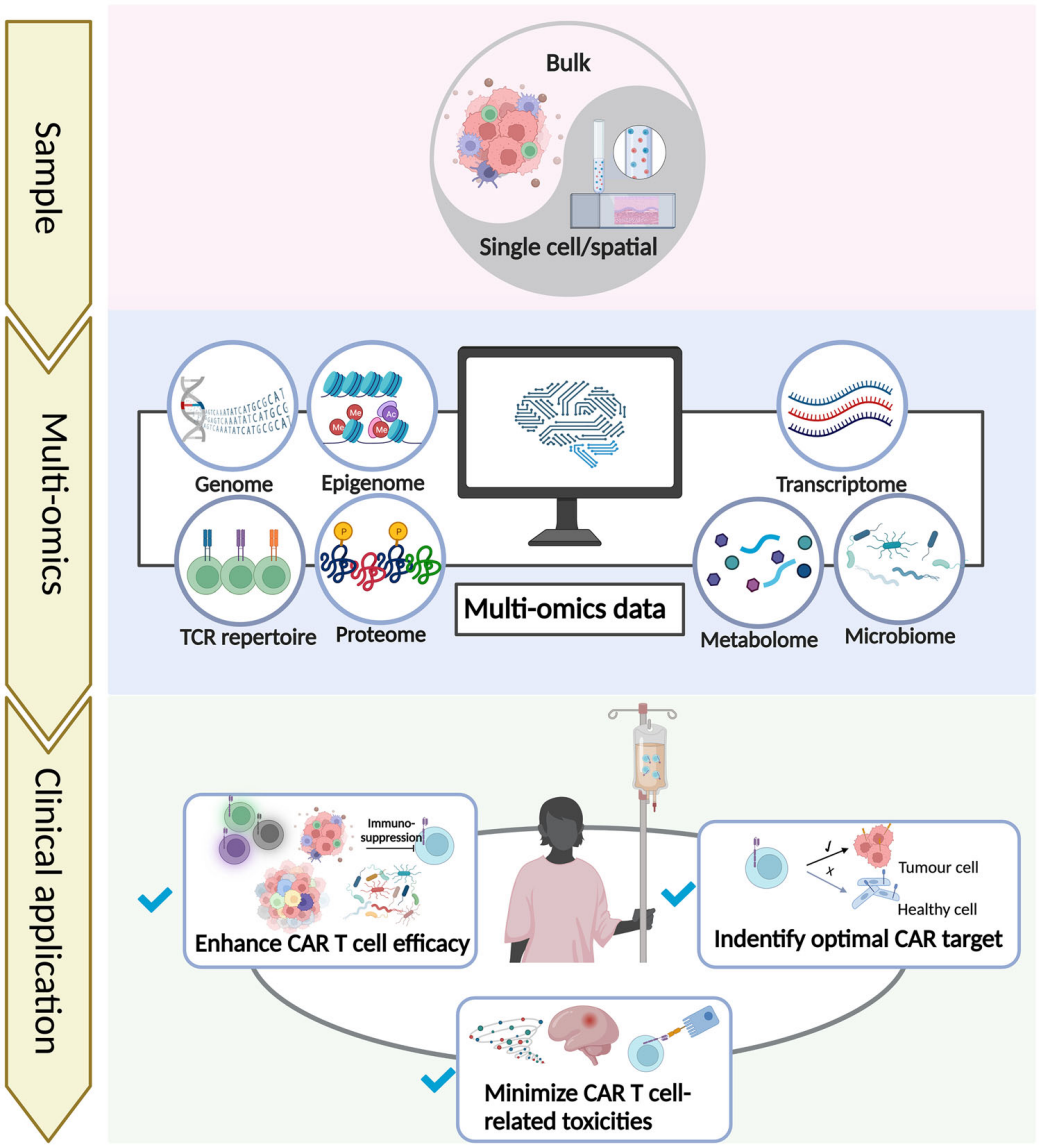
Applications of multi‐omics data in the context of CAR T cell therapy.

We first discussed various types of multidimensional profiling technologies, including genomics, epigenomics, transcriptomics, T cell receptor repertoire profiling, proteomics, metabolomics, and microbiomics, and further discussed how these data can be integrated and analyzed to advance our mechanistic understanding of CAR T cell therapies. We outlined studies leveraging multi‐omics data to characterize diverse features of malignant and non‐malignant cells, transitions in cell state, and various characteristics of the tumour microenvironment that are relevant to CAR T cell therapies. Of note, we also provided an overview of recent advances in spatial molecular profiling methods,^6^ which, although not yet been applied to CAR T cell research, hold great promise for advancing our understanding of the complex cellular interactions that govern the treatment outcome of CAR T cell therapy. Overall, our review represents a comprehensive summary of the potential of multidimensional omics data to enhance CAR T cell therapy for cancer treatment.

We discussed how genomics, transcriptomics, and/or proteomics data from both tumour and non‐malignant tissue can be applied to identify optimal targets, either single or combinatorial, with high levels of tumour specificity and coverage. Specifically, we emphasized the significance of utilizing single‐cell multi‐omics data to identify target antigens for CAR T cell therapies, aim to enhance antitumour efficacy and limiting toxicities. In a recent study, Kwon et al. constructed a single‐cell meta‐atlas comprising approximately 1.4 million cells of 17 cancer types and 12 normal organs from diverse sources and employed random forest and convolutional neural network methods to identify combinatorial (AND, OR and NOT) CAR targets that most effectively distinguish between individual malignant and normal cells.^7^ This study demonstrated how pan‐cancer and pan‐tissue expression data at the single‐cell resolution can be analyzed using machine learning algorithms to nominate CAR target pairs. Optimized computational approaches to integrate multi‐omics data would greatly facilitate the discovery and clinical evaluation of effective CAR target antigens.

We systematically summarized the molecular characteristics linked to the efficacy of CAR T cell therapy. These characteristics were identified through the integration of bulk and/or single‐cell multi‐dimensional omics data and can be classified into four main categories: T cell states and phenotypes, tumour cell characteristics, the tumour microenvironment and the microbiota. Our review provides a comprehensive summary of biological features associated with the response of CAR T cell therapy. Most recent studies have further demonstrated the promise of integrating multi‐dimensional omics data to understand the determinants of CAR T cell efficacy. For example, the efficacy of CAR T cells can be enhanced through the inhibition of key immunosuppressive factor in the tumour microenvironment (TME),^8^ and the composition of bacterial species of gut microbiome was also associated with the response to CAR T therapy.^9^

Finally, we summarized the factors related to CAR T cell‐induced toxicities revealed by muti‐omics data. These toxicities include cytokine release syndrome, immune effector cell‐associated neurotoxicity syndrome (ICANS) and on‐target, off‐tumour toxicities. Most recent studies have highlighted the power of multi‐omics technology in identifying novel biomarker of toxicity following CAR T cell therapy. For example, the integration of scRNA‐seq, scTCR‐seq and CITE‐seq demonstrated the expansion of Treg‐like cells on day 7 after infusion was found to be associated with reduced severe ICANS.^10^

In summary, our review provides a comprehensive overview of the multifaceted mechanisms that underlying the treatment outcomes of CAR T cell therapy through the integration of multi‐omics data. With the decreasing cost of omics technologies, particularly at single cell level, and the development of more robust data integration strategies, multidimensional omics data will play a crucial role in obtaining a complete molecular profile of CAR T cells. This advancement will accelerate the development of CAR T cell therapy, leading to improved efficacy and safety profiles, as well as better prediction and monitoring of patient responses.

## References

[1] Young RM , Engel NW , Uslu U , Wellhausen N , June CH . Next‐generation CAR T‐cell therapies. Cancer Discov. 2022;12(7):1625‐1633. doi:10.1158/2159-8290.cd-21-1683 35417527 PMC9262817

[2] Larson RC , Maus MV . Recent advances and discoveries in the mechanisms and functions of CAR T cells. Nat Rev Cancer. 2021;21(3):145‐161. doi:10.1038/s41568-020-00323-z 33483715 PMC8353572

[3] Amini L , Silbert SK , Maude SL , et al. Preparing for CAR T cell therapy: patient selection, bridging therapies and lymphodepletion. Nat Rev Clin Oncol. 2022;19:342‐355. doi:10.1038/s41571-022-00607-3 35318469

[4] Cappell KM , Kochenderfer JN . Long‐term outcomes following CAR T cell therapy: what we know so far. Nat Rev Clin Oncol. 2023;1‐13. doi:10.1038/s41571-023-00754-1 PMC10100620 37055515

[5] Yang J , Chen Y , Jing Y , Green MR , Han L . Advancing CAR T cell therapy through the use of multidimensional omics data. Nat Rev Clin Oncol. 2023;20:211‐228. doi:10.1038/s41571-023-00729-2 36721024 PMC11734589

[6] Moffitt JR , Lundberg E , Heyn H . The emerging landscape of spatial profiling technologies. Nat Rev Genet. 2022;23(12):741‐759. doi:10.1038/s41576-022-00515-3 35859028

[7] Kwon J , Kang J , Jo A , et al. Single‐cell mapping of combinatorial target antigens for CAR switches using logic gates. Nat Biotechnol. 2023. doi:10.1038/s41587-023-01686-y 36797491

[8] Zhang H , Yu P , Tomar VS , et al. Targeting PARP11 to avert immunosuppression and improve CAR T therapy in solid tumors. Nat Cancer. 2022;3(7):808‐820. doi:10.1038/s43018-022-00383-0 35637402 PMC9339499

[9] Stein‐Thoeringer CK , Saini NY , Zamir E , et al. A non‐antibiotic‐disrupted gut microbiome is associated with clinical responses to CD19‐CAR‐T cell cancer immunotherapy. Nat Med. 2023;29(4):906‐916. doi:10.1038/s41591-023-02234-6 36914893 PMC10121864

[10] Good Z , Spiegel JY , Sahaf B , et al. Post‐infusion CAR TReg cells identify patients resistant to CD19‐CAR therapy. Nat Med. 2022;28(9):1860‐1871. doi:10.1038/s41591-022-01960-7 36097223 PMC10917089

